# Comparative Proteomic Insights into the Lactate Responses of Halophilic *Salinicoccus roseus* W12

**DOI:** 10.1038/srep13776

**Published:** 2015-09-11

**Authors:** Hongyan Wang, Limin Wang, Han Yang, Yumeng Cai, Lifan Sun, Yanfen Xue, Bo Yu, Yanhe Ma

**Affiliations:** 1 CAS Key Laboratory of Microbial Physiological and Metabolic Engineering, Institute of Microbiology, Chinese Academy of Sciences, Beijing 100101, PR China; 2 State Key Laboratory of Microbial Resources, Institute of Microbiology, Chinese Academy of Sciences, Beijing 100101, PR China

## Abstract

Extremophiles use adaptive mechanisms to survive in extreme environments, which is of great importance for several biotechnological applications. A halophilic strain, *Salinicoccus roseus* W12, was isolated from salt lake in Inner Mongolia, China in this study. The ability of the strain to survive under high sodium conditions (including 20% sodium lactate or 25% sodium chloride, [w/v]) made it an ideal host to screen for key factors related to sodium lactate resistance. The proteomic responses to lactate were studied using W12 cells cultivated with or without lactate stress. A total of 1,656 protein spots in sodium lactate-treated culture and 1,843 spots in NaCl-treated culture were detected by 2-dimensional gel electrophoresis, and 32 of 120 significantly altered protein spots (fold change > 2, *p* < 0.05) were identified by matrix-assisted laser-desorption ionization time-of-flight mass spectrometry. Among 21 successfully identified spots, 19 proteins were upregulated and 2 were downregulated. The identified proteins are mainly involved in metabolism, cellular processes and signaling, and information storage and processing. Transcription studies confirmed that most of the encoding genes were upregulated after the cells were exposed to lactate in 10 min. Cross-protecting and energy metabolism-related proteins played an important role in lactate tolerance for *S. roseus* W12.

Lactic acid, as one of the three major organic acids, is widely used in the food and pharmaceutical industrials^1^. Its most common use is for the production of polylactic acid, a biodegradable polymer, which can substitute for conventional plastic materials. Microbial fermentation is the main method for the industrial production of lactic acid^2^. However, the main bottleneck in microbial lactic acid production is thought to be related to the inhibitory effects of the low pH of culture media on cell growth and viability and, thus, on lactic acid accumulation^3^. To counteract medium acidification, neutralizers, traditionally calcium carbonate, must be added to maintain a constant pH during fermentation^4^. The produced calcium lactate is then filtered to remove cells, decolored, evaporated, and acidified with sulfuric acid to convert the salt into lactic acid and insoluble calcium sulfate. The disadvantages of using calcium carbonate as a neutralizer include high sulfuric acid consumption and the large amount of waste byproduct (CaSO_4_) that is generated, which poses considerable economic and environmental problems^5^,^6^. Therefore, interest is emerging in developing clean fermentation processes for lactic acid production.

To overcome this environmental issue, efforts have been made to develop membrane-based separation and purification technologies, which do not produce similar salt-waste products. However, the utility of bipolar membranes is hindered by the intolerance of the membranes to multivalent cations, such as calcium ion^2^. Therefore, to employ bipolar membranes for lactic acid purification, monovalent ions such as Na^+^ must be used in the fermentation process. However, the production titers of the available lactic acid producers have not been satisfactory due to the high toxicity of Na^+^ to the strains^7^. The reason may be that neutralization of 1 mole lactic acid requires either 1 mole NaOH or only 0.5 mole CaCO_3_. Thus, the high ion concentration of sodium lactate may be a reason for stronger stress^8^. Alkaliphiles could maintain their cellular processes under high sodium conditions and the salt stress leads to the altered expression of specific and housekeeping genes^9^. Therefore, it has been suggested that alkaliphilic organisms, which are tolerant to salt, especially to monovalent ions such as Na^+^, may be promising for organic acid production^10^. In our previous study, the alkaliphilic *Exiguobacterium* sp. strain could produce L-lactate with NaOH as a neutralizing agent at pH 9.5, while the yield was low compared with industrial strains^11^. Subsequent experiments showed that this strain could not tolerate high sodium lactate concentrations (unpublished data). Although the alkaliphilic *Bacillus* sp. WL-S20 could produce >200 g/L of L-lactate^12^, the relatively low productivity and high fermentation pH also make this strain less applicable. Thus, the above findings partially indicate that response mechanisms between sodium lactate and sodium chloride may be different, even though the same cation is present.

Stress responses induce complex cellular reprograming. Observations suggest that acid stress adaptation overlaps with other stress responses, resulting in cross-protection. In *Salmonella typhimurium*, inactivation of the *atbR* (*pgi*) and *mviA* (*rssB*) genes, which regulate the expression of 10 acid-inducible proteins, increased the acid tolerance of host strain^13^. In *Saccharomyces cerevisiae*, the upregulation of genes controlled by the transcriptional factor Haa1, which is implicated in tolerance to other weak organic acids, serves an essential role related to lactic acid stress^14^. Although these mutants were not initially selected as stress resistant, the stress-related genes could be used to improve the performance of strains. Therefore, screening sodium lactate-tolerant strains and identifying key lactate-stress-related genes may be promising options for the development of efficient and clean lactic acid-production strategies by using NaOH as a neutralizer. Then, alkaliphile’s extraordinary physiological alkaline adaption capacity makes it a good source for stress-related gene isolation, which could be used to improve the performance of lactic-acid-fermenting strains. However, to date, no studies have been performed to examine sodium lactate responses in extremophiles.

In this study, a halophilic strain, *Salinicoccus roseus* W12, was isolated from soil samples of salt lake in Inner Mongolia, China. Our results showed that *S. roseus* W12 could grow well in media containing 20% (w/v) sodium lactate at pH 7.0 while could not consume sodium lactate. Comparative proteomic analyses and quantitative reverse transcription-PCR (RT-PCR) assays were performed with the aim of exploring the key factors related to lactate stress in strain *S. roseus* W12.

## Results

### Screening of high-concentration sodium lactate tolerant strains

The Inner Mongolia Plateau is a large plateau in the northern part of China. Owing to the dry climate, salts deposited during lake formation in the region. Thus, the salinity of lake brine mostly exceeds 10% (w/v, pH 8–9)^15^. Horikoshi medium containing 5% (w/v) NaCl was used as the base medium for salt-tolerant strain isolation^12^. Eighteen strains were isolated from soil samples in salt lake in Inner Mongolia. After inoculation in medium containing 15% (w/v) sodium lactate instead of NaCl, only 3 viable strains emerged. To further isolation of high lactate-tolerance strain, Horikoshi medium containing 20% (w/v) sodium lactate was used, and growth of all 3 strains was observed. The growth characteristics of the isolated strains are summarized in Table 1. According to their 16S rRNA gene sequences, the above three strains were tentatively identified as *Halobacillus trueperi*, *Halomonas salifodinae*, and *S. roseus*. *S. roseus* and *H. trueperi* stains had same OD_600_ value (0.19 ± 0.01 and 0.18 ± 0.01, respectively) at 6 h, while higher OD_600_ value was observed in *S. roseus* strains (1.01 ± 0.03) than that in *H. trueperi* strains (0.89 ± 0.04) at 12 h. Compared with the other two strains, a higher optical density (OD_600_ = 2.12 ± 0.15) was obtained with the *S. roseus* seeded at equivalent densities with the other two strains when grown in high sodium lactate-medium after 24 h. Then *S. roseus* strain designated as W12 was used for subsequent studies.

**Figure 1:**
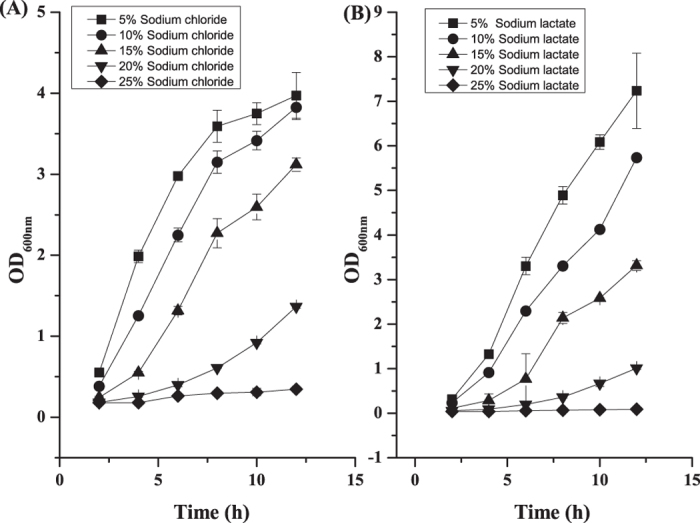
Effects of salt stress on the cell growth of *Salinicoccus roseus* W12. (**A**) Effects of NaCl on cell growth. (**B**) Effects of sodium lactate on cell growth. Strains were grown in Horikoshi medium to mid-log phase and transferred (1%, v/v) to media containing the indicated concentrations of NaCl and sodium lactate. The OD_600_ of the cultures were monitored periodically for 12 h. Experiments were performed in triplicate. Error bars represent the standard deviations of the means of three independent experiments.

To further investigate the effects of salt stress on cell growth, W12 cells were inoculated into medium containing different concentrations of NaCl and sodium lactate. *S. roseus* W12 grew well on medium containing 5% (w/v) and 10% (w/v) NaCl, while a high NaCl concentration (20%, w/v) inhibited cell growth. Increasing NaCl concentrations (higher than 25%, w/v) retarded cell growth (Fig. 1A). No growth of the W12 strain was observed when the NaCl concentration was set at zero, indicating that *S. roseus* W12 is a halophile. Compared with NaCl, higher sodium lactate concentrations resulted in lower OD_600_ values. After 12 h of cultivation, the OD_600_ value decreased from 7.23 ± 0.84 in 5% (w/v) sodium lactate to 0.09 ± 0.02 in 25% (w/v) sodium lactate (Fig. 1B). There was no obvious consumption of lactate in any of the cultures, suggesting that the observed lactate tolerance was not due to lactate utilization by strain W12.

### Growth characteristics of *S. roseus* W12 under lactate or NaCl stress

**Figure 2:**
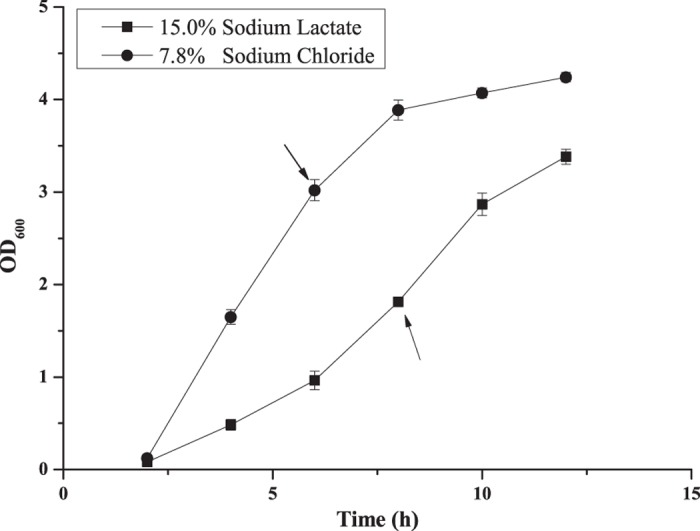
Growth of *S. roseus* W12 under lactate or NaCl stress with the same Na^+^ concentration. The cells were cultivated in medium (pH 7.0) supplemented 15% sodium lactate or 7.8% sodium chloride (w/v), which contained the same molar concentrations of free Na^+^ ions. The OD_600_ of the cultures were monitored periodically for 12 h. The arrows indicate the sampling times used for proteomic-analysis studies.

Based on the results shown in Fig. 1, the growth curves of *S. roseus* W12 on media containing NaCl and sodium lactate were different. Cell growth on medium containing the same Na^+^ concentration (7.8% [w/v] NaCl and 15% [w/v] sodium lactate, respectively) was investigated to confirm this observation. Strain W12 grew much faster under NaCl stress than under lactate stress (Fig. 2). The OD_600_ value of *S. roseus* W12 exposed to NaCl reached 3.02 ± 0.12 after 6 h, while that in medium containing sodium lactate reached only 0.96 ± 0.10 during the same time. Under sodium lactate-stress conditions, the growth of *S. roseus* W12 was slowed and the OD_600_ value reached 1.81 ± 0.04 by 8 h-cultivation, which corresponded to the middle of the exponential growth phase. Therefore, lactate stress exerted a significant inhibitory effect on the growth of *S. roseus* W12 with the same Na^+^ concentration. Cells in 7.8% NaCl grows well in the first 12-h incubation, and OD value reached 4.24 ± 0.05 at 12 h, which is consistent with the cells growing in 5% (OD_600_ = 3.97 ± 0.28) and 10% NaCl (OD_600_ = 3.83 ± 0.15), indicating the predominant lactate-tolerant capacity of *S. roseus* W12. Cells in the respective mid-exponential growth (6 h in NaCl or 8 h in lactate) were collected for comparative proteomic analyses.

### Protein expression alterations in response to lactate stress

**Figure 3:**
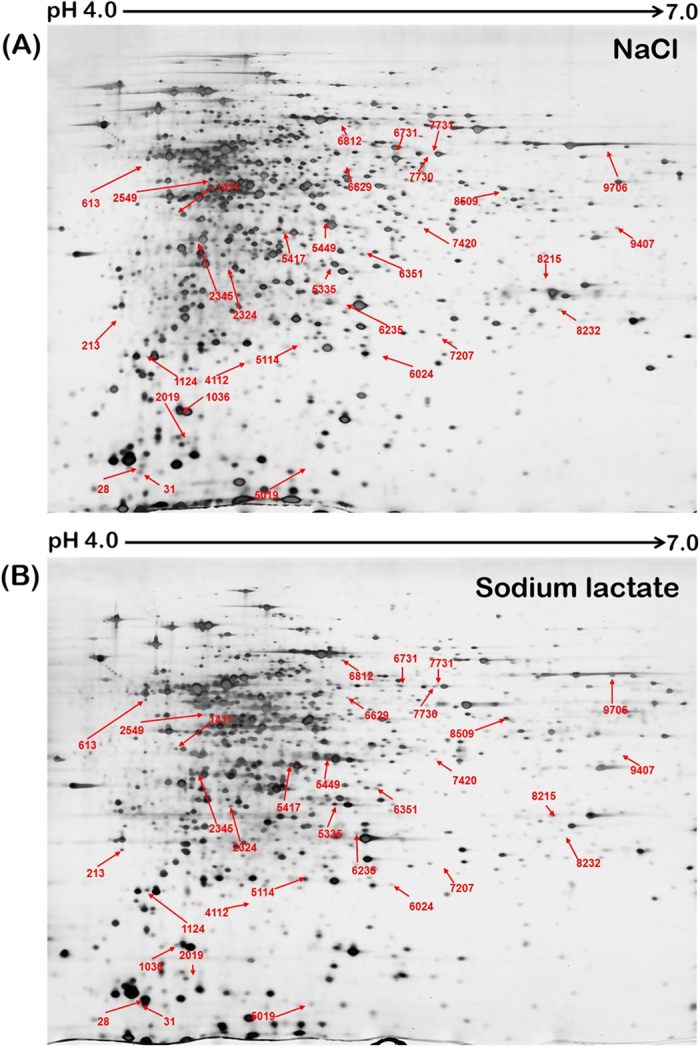
Comparative proteomic profiling of *S. roseus* W12 exposed to (A) 7.8% (w/v) NaCl or (B) 15% (w/v) sodium lactate. The differentially expressed proteins were labeled with spot numbers. Experiments were performed in triplicate.

The global proteomic response of *S. roseus* W12 to lactate stress was profiled by 2-D gel electrophoresis and three technical replicates (same samples analyzed multiple times) were carried out for each treatment. The cells collected from cultures incubated with 7.8% (w/v) NaCl were used as the control group (strain W12, lactate^−^), and those collected from cultures incubated with 15% (w/v) sodium lactate were used as the experimental group (strain W12, lactate^+^). In 3 independent experiments, the protein abundances were significantly altered (fold change > 2, *p* < 0.05) in 120 of approximately 1,656 spots from the lactate^+^ cultures and 1,843 spots from the NaCl culture (Fig. 3), suggesting that the presence of lactate affected the cellular physiology. Among these, 26 spots were upregulated and 94 spots were downregulated in lactate^+^ cultures. Thirty-two spots (26 upregulated spots and 6 downregulated spots) with a >2.0-fold difference in abundance on 2-D gels were collected and digested with enzyme. MALDI-TOF-TOF-MS was employed for mass spectrometric identification. Twenty-one spots were successfully identified. Two proteins were identified as the same protein (Table 2). The protein content in the remaining spots was either too low for valid analysis or overlapped with those of other spots on the gels.

To fully identify the spots, the whole genome of *S. roseus* W12 was sequenced, and annotated with the NCBI Prokaryotic Genomes Automatic Annotation Pipeline, and functionally annotated using the Clusters of Orthologous Genes (COG) and KEGG databases. The genome sequence of *S. roseus* W12 obtained by using the Illumina HiSeq 2000 system, which was performed by the Chinese National Human Genome Center at Shanghai, China. Through the data assembly, we obtained 15 scaffolds (26 contigs), and the average length of scaffold is 171 Kb with a total length of 2.56 Mb. The draft genome sequence was deposited in DDBJ/EMBL/GenBank under accession number JXII00000000. Based on the genomic information, the identified protein spots in 2-D gels were also targeted to its gene sequence, as shown in Table 2.

**Figure 4:**
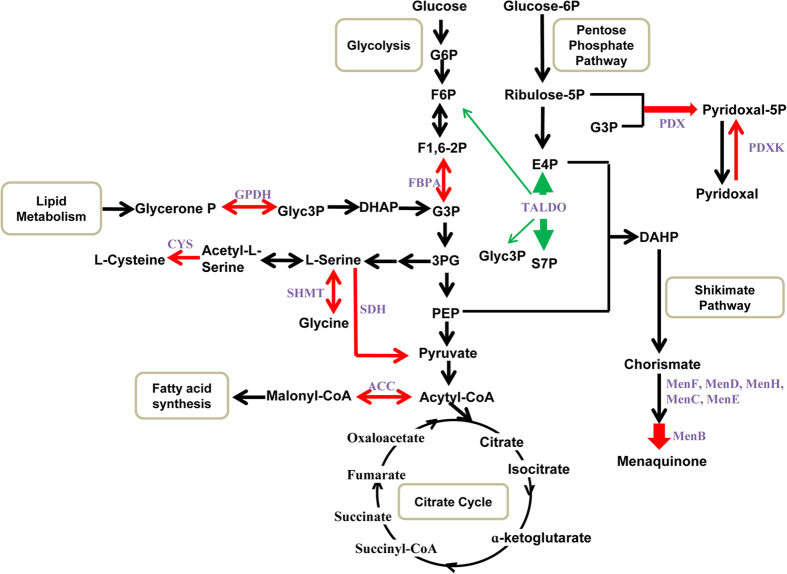
Schematic overview of the metabolic pathways associate with the differentially expressed proteins identified in *S. roseus* W12 cells exposed to lactate stress. Red arrows indicate upregulated proteins, and green arrows indicate downregulated proteins. The arrow widths indicate the relative expression levels of proteins, which are expressed quantitatively in Table 2. *G6P*, glucose 6-phosphate; *F6P*, fructose 6-phosphate; *F1,6-2P*, fructose 1,6-bisphosphate; *G3P*, glycerate 3-phosphate; *3PG*, 3-phosphoglycerate; *PEP*, phosphoenolpyruvate; *DAHP*, 3-deoxy-D-arabino-heptulosonate 7-phosphate; *Glyc3P*, glyceraldehyde 3-phosphate; *G6P*, glucose 6-phosphate; *E4P*, erythrose 4-phosphate; *S7P*, sedoheptulose 7-phosphate; *DHAP*, dihydroxyacetone phosphate.

All differentially expressed proteins were assigned to COG functional categories and grouped into 4 functional categories: cellular processes and signaling (D/O; 2 proteins), information storage and processing (J; 1 protein), metabolism (C/E/F/G/H/I; 15 proteins), and poorly characterized (R; 2 proteins) (Table 2). Notably, approximately 76% of the identified proteins belonged to the metabolism category, including the most significantly upregulated protein (spot number 7420 with a 40-fold increase). Two spots (7731 and 7730) were identified as serine hydroxymethyltransferase (SHMT; EC 2.1.2.1), and lactate stress induced 3.23–3.87 increase in SHMT expression. The results are summarized in Fig. 4, which enabled us to better understand the contribution of protein expression to the metabolic shift. Notably, many enzymes involved in amino acid transport and metabolism, including pyridoxal biosynthesis protein (PDX), SHMT, cysteine synthase (CYS), pyridoxal kinase (PDXK), and serine dehydratase (SDH), as well as other enzymes involved in carbohydrate transport and metabolism (phosphocarrier protein [HPR], uridylyltransferase [ULT], and fructose-1,6-bisphosphate aldolase [FBPA]) and lipid transport and metabolism (dihydroxynaphthoic acid synthetase [MenB] and acetyl-CoA carboxylase biotin carboxylase [ACC]) were upregulated in W12 lactate^+^ cells. In addition, transaldolase (TALDO, spot number 7207) involved in the non-oxidative phase of the pentose phosphate pathway was significantly downregulated with a 3,533-fold decrease.

### Gene transcription-level alterations in response to lactate shock

At first, the sampling time for real-time PCR (RT-PCR) was same as 2D gels analysis. However, most of the genes showed no detectable induction after prolonged stress exposure, which is inconsistent with the data from 2D (data not shown). It has been reported that differential mRNA expression levels can revert back following prolonged stress exposure^16^. Furthermore, from the biological point of view, mRNA level represents the intermediate state of gene expression while protein levels are affected by both factors that influence transcript levels and those that affect later stages of expression^17^. That might be the reason that the data from RT-PCR analysis were inconsistent with those from 2D gel analysis. On the other hands, if the gene expression level varied in the initial time at which the high concentration of lactate was just added, it should be a good way to solidly confirm the functions. Then the short time exposure was conducted to investigate the differential mRNA expression levels to see whether the identified proteins are indeed involved in the response to lactate stress.

**Figure 5:**
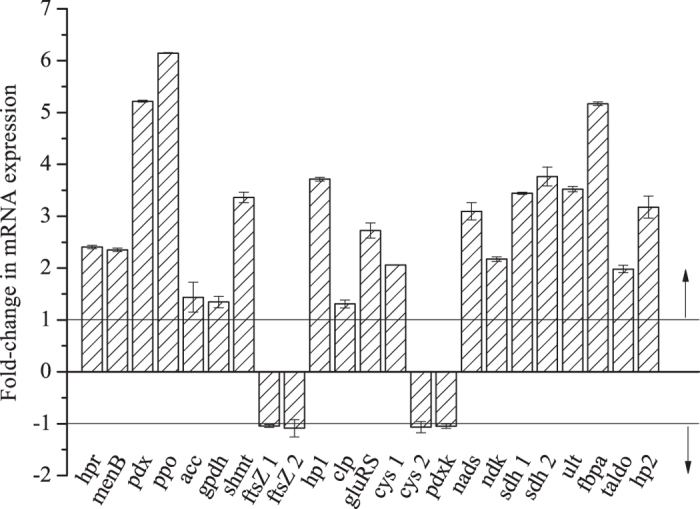
Determination of the relative transcriptional levels of genes encoding upregulated/downregulated proteins under lactate stress by RT-PCR. The y-axis indicates the fold-changes in genes in stressed lactate^+^ cells compared to lactate^–^ cells. The error bars represent the standard deviations of the means of three independent experiments.

Cells cultivated without lactate stress were shocked by 15% (w/v) lactate in 10 min. Subsequently, the transcriptional levels of above-mentioned genes were compared to those of the non-stressed cells. According to the NCBI database, each of cell division protein (FTSZ), CYS, and SDH proteins are encoded by 2 genes. So, there were totally 23 genes for RT-PCR analysis. As shown in Fig. 5, the transcriptional levels 19 of the 23 genes were increased after 10 min of lactate stress. Gene expression differences of *ftsZ1/ftsZ2* (encoding the cell division protein FtsZ), *cys2* (encoding cysteine synthase, EC 2.5.1.47) and *pdxk* (encoding pyridoxal kinase, EC 2.7.1.35) were not obviously in W12 lactate^+^ cells. The transcriptional alterations observed for 17 genes agreed with the protein-expression differences observed on 2-DE gels, while the transcriptional levels of 6 genes (*ftsZ1/ftsZ2*, *cys2*, *pdxk*, *taldo*, and *hp2*) differed when compared to the corresponding protein-expression levels observed. The highest fold change (6.15-fold) in stressed lactate^+^ cells compared to lactate^−^ cells was observed in the *ppo* gene (encoding protoporphyrinogen oxidase, EC 1.3.3.4).

## Discussion

Extremophiles can survive in extreme environments, which is very close to the industrial production conditions. Therefore, the adaptation of extremophiles to harsh environmental conditions enables several important applications in biotechnology. Previous studies have led to the discovery of many types of novel enzymes and other valuable products, such as antibiotics, bioactive compounds, carotenoids, and siderophores, which exhibit interesting properties^18^. Recently, a growing trend for organic acid production by extremophiles has emerged^11^,^12^. Some extremophiles are salt tolerant and cannot grow in the absence of sodium or other monovalent ions, and hence bases such as NaOH can be used to maintain the pH of the production media instead of CaCO_3_. From a biotechnological point of view, screening the key proteins and their encoding genes from extremophiles, such as halophiles, seems more attractive for identifying factors that can be introduced into industrial strains to increase their salt tolerances at neutral pH, thus reducing the base costs. In this study, a halophilic, non-lactate utilizing *S. roseus* strain was chosen as a candidate to explore the mechanism of sodium lactate resistance. *S. roseus* was first isolated from a solar saltern in Alicante, Spain^19^. It is a Gram-positive, red-pigmented, and moderately halophilic species that grows at 15–40 °C and pH 6–9 (optimal growth occurs at 37 °C and pH 7.5–8.0). Studies showed that no growth occurred in media without NaCl, and the minimum NaCl concentration required by this strain was 0.9% (w/v)^20^. Although the *S. roseus* strain W12 isolated in this study is not a lactic acid producer, the characteristic of both lactate and NaCl tolerance makes it an ideal model to screen for key factors related to sodium lactate stress.

By performing comparative proteomic analyses, 21 spots were successfully identified by 2-D gel and MALDI-TOF detection. Among these, 19 spots were upregulated and 2 spots were downregulated. The identified proteins are mainly involved in metabolism, cellular processes and signaling, and information storage and processing (Fig. 4). Proteins involved in amino acid biosynthesis, such as SHMT (encoded by *shmt*), serine dehydratase (SDH, EC 4.3.1.17; encoded by *sdh*), and cysteine synthase (CYS, EC 2.5.1.47; encoded by *cys*) were upregulated in lactate^+^ cells. SHMT catalyzes the inter-conversion of serine to glycine^21^. Previous results showed that SHMT was salt-induced and increased the level of glycine betaine via serine and choline, which could in turn balance the external osmolality^22^. For lactate^+^ cells, SHMT expression at the protein and transcription levels was increased by 3.87 and 3.36-fold, respectively. SHMT upregulation may enable cells to produce more glycine following exposure to high salt concentrations. SDH is a member of the β-family of pyridoxal phosphate-dependent enzymes and catalyzes the deamination of serine to pyruvate. The SDH enzyme functions by lowering the activation energy for converting serine into pyruvate^23^. Moreover, we found that CYS was upregulated in lactate^+^ cells. CYS catalyzes the conversion of acetyl-L-serine to L-cysteine. The observed upregulation of SDH and CYS indicates that increased serine can be produced under lactate stress. *S. roseus* W12 may use serine-metabolism strategies to adapt to lactate stress.

Lactate stress induced significant changes (40-fold) in the expression of dihydroxynaphthoic acid synthetase (MenB, EC 4.1.3.36; encoded by *MenB*). MenB catalyzes the sixth reaction in the synthesis of menaquinone (vitamin K2) (Fig. 4). Vitamin K is a quinone that serves as an electron transporter during respiration, enabling bacteria to generate the energy required for survival^24^. Upregulation of acetyl-CoA carboxylase (ACC, EC 6.4.1.2; encoded by *acc*) was observed in lactate^+^ cells. ACC is a biotin-dependent enzyme that catalyzes the irreversible carboxylation of acetyl-CoA to produce malonyl-CoA. The most important function of ACC is to produce the malonyl-CoA substrate for the biosynthesis of fatty acids^25^. Fatty acids biosynthesis is essential for cell growth and viability, which produces energy for cells during the citric acid cycle. Microbial adaptation to osmotic pressure is an energy-consuming process^26^, and the lactate-induced upregulation of MenB and ACC might enable cells to adapt to harsh conditions.

Proteins involved in pyridoxal biosynthesis, such as pyridoxal biosynthesis protein (PDX, EC 4.3.3.6; encoded by *pdx*) and pyridoxal kinase (PDXK, EC 2.7.1.35; encoded by *pdxk*) were upgraded in lactate^+^ cells. PDX catalyzes the formation of pyridoxal 5′-phosphate (pyridoxal-5P) from ribose 5-phosphate (RBP), glyceraldehyde 3-phosphate (G3P), and ammonia (Fig. 4). PDXK catalyzes the conversion of pyridoxal (vitamin B6) to pyridoxal-5P. Previous experiments showed that *pdx* deletion mutants were hypersensitive to osmotic and oxidative stress^27^, indicating that PDX played an indirect role in the resistance to stress.

Lactate stress also induced the upregulation of glycerol-3-phosphate dehydrogenase (GPDH, EC1.1.5.3, encoded by *gpdh*) at both the protein level (4.03-fold) and the transcription level (1.34-fold). GPDH serves as a major link between carbohydrate metabolism and lipid metabolism, playing a major role in lipid biosynthesis. Through the reduction of dihydroxyacetone phosphate to glycerol 3-phosphate, GPDH facilitates the prompt dephosphorylation of glycerol 3-phosphate to form glycerol. Considering that glycerol is a well-known compatible solute that may counteract osmotic pressure in cells, the elevated glycerol level observed was likely an artifact caused by the presence of lactate^28^.

Moreover, we found that transaldolase (TALDO, EC 2.2.1.2; encoded by *taldo*) was significantly downregulated by 3,533-fold at the protein level under lactate stress conditions while RT-PCR results showed that *taldo* transcription was a little bit upregulated (1.98-fold) when cells were exposed to the lactate stress in the initial 10 min. TALDO creates a reversible link between the pentose pathway and glycolysis (Fig. 4), which allows cells to adapt NADPH and ribose-5-phosphate production to meet their immediate needs^29^. Several studies have reported that TALDO activity is rate-limiting for xylose fermentation in recombinant *Saccharomyces cerevisiae*^30^,^31^. TALDO downregulation in lactate^+^ cells suggests that pentose metabolism is impaired during lactate stress. Surprisingly, fructose-1,6-bisphosphate aldolase (FBPA, EC 4.1.2,13, encoded by *fbpa*), which is involved in glycolysis, was upregulated, suggesting a potential shift in carbon utilization from the pentose pathway to glycolysis. The use of the most efficient sugar-metabolism pathway (glycolysis) ensures that W12 lactate^+^ cells can access energy readily when harsh conditions are encountered. It was also reported that differential mRNA expression levels can revert back following prolonged stress exposure^32^. Peng *et al.* reported that 4 tested *E. coli* strains displayed similar *cadA* (encoding lysine-dependent decarboxylase) induction after 15 min of organic acid stress, while no induction of this gene was detectable after prolonged stress exposure^16^. In this study, sodium lactate-induced and NaCl-induced changes in the protein profiles of *S. roseus* W12 were examined using cells in exponential growth phase. From biological point of view, mRNA level represents the intermediate state of gene expression. Protein levels are affected by both factors that influence transcript levels and those that affect later stages of expression. The transcription results and protein abundance measurement may be inconsistent^33^, which gives the explanation of our RT-PCR analysis of the exponential growth phase samples. Notably, most of the encoding genes of identified proteins were upregulated after the cells were exposed to lactate shock in 10 min, which provided the solid evidence to support the results from 2D gels.

In conclusion, the results indicated that genes involved in metabolism, cellular processes and signaling, and information storage and processing were regulated in response to lactate stress. Some of the identified proteins are known to serve osmoadaptive functions (e.g., SHMT, GPDH, PDX, PDXK, and MenB). Notably, most of these proteins play a more general role in cross-protecting cells against diverse stress conditions. Furthermore, several of the implicated proteins are involved in energy metabolism, such as TALDO, FBPA, and ACC. Based on the above results, two possible mechanisms for lactate tolerance in *S. roseus* W12 were speculated. First, the accumulation of osmotically active compatible proteins, such as SHMT, GPDH, PDX and PDXK, is used by *S. roseus* W12 to ease growth under stress conditions and also to stabilize macromolecules against lactate tress. Second, *S. roseus* W12 tend to use the most effective energy metabolism pathway to counteract the large energy-consuming under lactate stress. Cross-protecting proteins and energy-metabolism-related proteins played important role in the lactate tolerance of *S. roseus* W12, highlighting potential tools for generating lactic acid-producing strains in clean fermentation processes in the future. Confirmation of the functions of the identified genes in lactic acid-fermenting strains is currently under investigation.

## Methods

### Isolation of salt-tolerant strains

Soil samples were collected from salt lakes in Inner Mongolia, China. Approximately 2 g of each sample was enriched for 3 h in 50 ml of Horikoshi medium at 37 °C, with shaking at 180 rpm. An aliquot of incubation was plated on Horikoshi agar medium containing 2% (w/v) agar. After a 24-h incubation period at 37 °C, representative colonies were selected based on their colors and shapes. Then, the selected colonies were incubated in selective medium to monitor the growth rates.

The Horikoshi medium contained (w/v): 1% glucose, 0.5% yeast extract, 0.5% polypeptone, 5% NaCl, 0.1% K_2_HPO_4_, and 0.02% Mg_2_SO_4_·7H_2_O. The pH was adjusted to 7.0 by adding sterilized 10% Na_2_CO_3_ (w/v). The selective medium contained (w/v): 1% glucose, 0.5% yeast extract, 0.5% polypeptone, 0.1% K_2_HPO_4_, and 0.02% Mg_2_SO_4_·7H_2_O. Different concentrations of sodium lactate (ranging from 5% to 25%, w/v) were added to the medium and the pH was adjusted to 7.0 by NaOH solution before sterilization.

### Cultivation conditions with or without sodium lactate stress

The best grown colony (designated W12) was selected for further study. Strain W12 was maintained on a Horikoshi agar slant. A loop of cells from the fully grown slant was inoculated into 5 ml Horikoshi media in 15 ml-conical flasks and incubated for 12 h at 37 °C at 180 rpm. Then, the seed culture was inoculated in medium with or without sodium lactate stress (1% inoculum). W12 cells grown in medium with or without lactate were designated as lactate^+^ and lactate^−^ cells, respectively.

The composition of the medium containing lactate was (w/v): 1% glucose, 0.5% yeast extract, 0.5% polypeptone, 15% sodium lactate, 0.1% K_2_HPO_4_, and 0.02% Mg_2_SO_4_·7H_2_O. The composition of the medium not containing lactate was (w/v): 1% glucose, 0.5% yeast extract, 0.5% polypeptone, 7.83% NaCl, 0.1% K_2_HPO_4_, and 0.02% Mg_2_SO_4_·7H_2_O. The media were autoclaved at 115 °C for 15 min, and the pH was adjusted to 7.0. To ensure that the changes in protein profiles resulted from lactate treatment, the molar Na^+^ concentration used in both media was the same, and lactate^−^ W12 strain cells were run in parallel with lactate^+^ W12 samples.

### Preparation of protein extracts and comparative proteomic analysis

Strain W12 lactate^+^ and lactate^−^ cells in exponential growth phase (optical density of OD_600_ = 1.81 and OD_600_ = 3.02, respectively) were chilled and harvested from 50-ml cultures by centrifugation at 4,000 × *g* for 10 min at 4 °C. The cell pellets were frozen in liquid nitrogen and stored at −80°C until proteome analysis was performed.

Samples were suspended in lysis buffer (PL039; Sangon Biotech, Shanghai, China). Cells were lysed by sonication at 80–100 W for 3 min, and the debris was removed by centrifugation at 12,380 × *g* and 4 °C for 30 min. Subsequently, the supernatant was incubated overnight with 1 ml ice-cold acetone at −20 °C to precipitate the proteins. After centrifugation at 12,380 × *g* and 4 °C for 30 min, the precipitated proteins were air dried and solubilized in sample lysis buffer (PL039). Total protein concentrations were determined using the Non-Interference Protein Assay Kit (Sangon Biotech, Shanghai, China).

Approximately 150 μg of protein from various W12 strain samples was solubilized in solubilization buffer and resolved on Immobilized pH Gradient (IPG) Strips (pH 4–7, nonlinear; GE Healthcare, USA). First-dimension isoelectric focusing (IEF) was conducted at 20 °C with an Ettan IPGphor system (GE healthcare, USA). Focusing was performed in 5 steps: 50 V for 12 h, 500 V for 1 h, 1,000 V for 1 h, 1,000–10,000 V for 8 h, and a final phase of 10,000 V for 11 h. After equilibrium was reached, the IPG strips were placed on the top of vertical 12.5% polyacrylamide gels, and sodium dodecyl sulfate polyacrylamide gel electrophoresis (SDS-PAGE) was performed at 15 °C. Electrophoresis was performed at 100 V for 45 min followed by 200 V until the bromophenol blue dye reached the bottom of the gels. Protein spots on the gels were visualized by silver staining. Each set of samples was independently analyzed in triplicate.

### Analyses of 2-dimension gels

Two-dimensional (2-D) gels were scanned at a resolution of 300 dots per inch (dpi) by an Image Scanner III (GE healthcare, USA), and 2-DE gel image-analysis software PDQuest^TM^ Basic, version 8.0.1 (Bio-Rad) was used for gel analysis. After background subtraction and spot detection, spots were matched. Spot quantification in the control and sodium lactate treated gels was done by measuring spot volumes (intensity × mm^2^). Statistically significant differences were discerned by performing Student’s *t* test. Proteins spot showing significant changes in up-regulation and down-regulation were extracted, digested, rehydrated, and identified in an Applied Biosystems MALDI-TOF/TOF 4700 proteomic analyzer (Applied Biosystems, Framingham, MA). Protein annotations were extracted from the National Center for Biotechnology Information (NCBI) databases and the Kyoto Encyclopedia of Genes and Genomes database (KEGG; http://www.genome.jp/kegg/) by a MASCOT 2.0 search engine (Matrix Science, London, United Kingdom).

### Analysis of gene expression by quantitative RT-PCR

To evaluate the expression of genes encoding significantly upregulated and downregulated proteins at the transcription level, RT-PCR was performed using Power SYBR Green PCR Master Mix (Applied Biosystems, CA). The exposure of *S. roseus* W12 to NaCl and lactate stress conditions was conducted as previously described^16^. Briefly, single colonies were inoculated into 5 mL Horikoshi broth and grown for 12 h at 37 °C and 180 rpm. The primary cultures were diluted 1:50 into 50 mL Horikoshi broth and grown for 8 h at 37 °C and 180 rpm until they reached exponential growth phase. Aliquots of 10 mL cultures were subsequently centrifuged for 5 min at 5,000 × *g*. Recovered cells were resuspended in either Horikoshi broth with 7.8% (w/v) NaCl (named as W12 lactate^−^), or Horikoshi broth with 15% (w/v) sodium lactate (named as W12 lactate^+^). After a 10-min incubation at 37 °C, samples were taken from different broths, and RNA isolation and RT-PCR analysis were performed as described previously^34^. Strain W12 lactate^+^ and lactate^–^ cells exposed or not exposed to lactate stress were harvested by centrifugation (5,000 × *g* for 10 min, 4 °C) for RNA isolation using the E.Z.N.A.^TM^ Bacterial RNA Kit (Omega Bio-tek, USA). Total RNA concentration was determined from the absorbance at 260 nm (NanoVue, GE Healthcare). Using appropriate gene-specific primers (Table 3), cDNA copies were prepared with the FastQuant RT Kit (with gDNase) (Tiangen, China) and amplified with SYBR Premix Ex Taq. The 2^–△△Ct^ method was used for the relative quantification of mRNA levels, and 16S rRNA was used as the internal reference. The threshold cycle for each of the PCR reaction with different concentrations of cDNA was determined and compared against a DNA standard that was run in parallel. From these results, the relative expression levels of gene-specific mRNAs in the samples were calculated. Results are reported as the average of at least 3 experiments and showed a variation of less than 15%.

## Additional Information

**How to cite this article**: Wang, H. *et al.* Comparative Proteomic Insights into the Lactate Responses of Halophilic *Salinicoccus roseus* W12. *Sci. Rep.* **5**, 13776; doi: 10.1038/srep13776 (2015).

## Figures and Tables

**Table 1:** Growth characteristics of the isolated strains※.

**Medium**	**1**	**2**	**3**	**4**	**5**	**6**	**7**	**8**	**9**	**10**	**11**	**12**	**13**	**14**	**15**	**16**	**17**	**18**
Horikoshi	+	+	+	+	+	+	+	+	+	+	+	+	+	+	+	+	+	+
15% sodium lactate^a^	−	−	+	−	−	−	−	−	−	−	−	+	−	+	−	−	−	−
20% sodium lactate^b^	−	−	+	−	−	−	−	−	−	−	−	+	−	+	−	−	−	−

^※^ +, colony detected; −, no colony detected.

^a^Horikoshi medium containing 15% (w/v) sodium lactate, instead of 5% (w/v) NaCl at pH 7.0.

^b^Horikoshi medium containing 20% (w/v) sodium lactate, instead of 5% (w/v) NaCl at pH 7.0.

**Table 2:** Differentially expressed proteins following exposure to lactate stress.

COGclass^a^	SSP^b^	**Function**	Fold change(Sodium lactate*vs.* NaCl)^c^	***p* value**^d^	GenBankAccessionNo.^e^
I	7420	Dihydroxynaphthoic acid synthetase (MenB)	+40.12	0.026	KIH69967
E	5449	Pyridoxal biosynthesis protein (PDX)	+9.65	0	KIH70410
H	9706	Protoporphyrinogen oxidase (PPO)	+9.60	0.047	KIH69896
I	6731	Acetyl-CoA carboxylase biotin carboxylase (ACC)	+4.11	0.002	KIH72052
C	6629	Glycerol-3-phosphate dehydrogenase (GPDH)	+4.03	0.019	KIH71530
E	7731/7730	Serine hydroxymethyltransferase (SHMT)	+3.87/3.23	0.011/0.033	KIH70495
D	613	Cell division protein FtsZ (FtsZ)	+3.72	0.006	KIH71107
R	213	Hypothetical protein	+3.71	0.012	KIH71119
O	5114	Clp protease (CLP)	+3.69	0.006	KIH71630
J	6812	Glutamyl-tRNA synthetase (GLURS)	+3.41	0.008	KIH70385
E	5417	Cysteine synthase (CYS)	+3.17	0.028	KIH70417
E	5335	Pyridoxal kinase (PDXK)	+2.81	0.004	KIH70353
C	1411	NAD synthetase (NADS)	+2.60	0.004	KIH70220
G	28	Phosphocarrier protein (HPR)	+2.59	0	KIH69853
F	1036	Nucleoside diphosphate kinase (NDK)	+2.57	0.018	KIH71947
E	2324	Serine dehydratase (SDH)	+2.50	0.026	KIH71066
G	8509	Uridylyltransferase (ULT)	+2.44	0.025	KIH71160
G	2345	Fructose-1,6-bisphosphate aldolase (FBPA)	+2.23	0.027	KIH70763
G	7207	Transaldolase (TALDO)	−3533.10	0.017	KIH70504
R	8232	Hypothetical protein	−52.06	0.026	KIH70008

^a^*C*: Energy production and conversion; *D*: cell cycle control, cell division, chromosome partitioning; *E*: Amino acid transport and metabolism; *F*: Nucleotide transport and metabolism; *G*: Carbohydrate transport and metabolism; *H*: Coenzyme transport and metabolism; *I*: Lipid transport and metabolism; *J*: Translation, ribosomal structure and biogenesis; *O*: Post-translational modification, protein turnover, and chaperone functions; *R*: general function prediction only.

^b^Reference to the protein numbering shown in Fig. 3.

^c^Fold-changes in protein expression were calculated using the “strain W12 lactate^−^ gel” as a reference. “+” up-regulated protein spots. “−” down-regulated protein spots.

^d^The significant level of the test is 5%.

^e^The accession numbers shown refer to the genes in the draft genome sequence of *Salinicoccus roseus* W12 deposited in the NCBI under accession number JXII00000000.

**Table 3:** Primers used for RT-PCR analysis.

**Gene (SSP)**^a^	**Forward primer (5′ ⟶ 3′)**	**Reverse primer (5′ ⟶ 3′)**	**Up/Downregulated**
*menB* (7420)	GAGTTCTACAACGGCATCGCCAAAG	AGGAACCTCATCTTCACCGACATAG	Up
*pdx* (5449)	CGTAGTCCTGATAGTGGGTGG	AGGCAACATCGTGGAGGCGGTAAGG	Up
*ppo* (9706)	GATTATCCGCAGCCCATTAC	GTATGATGTCCCCGTCTGGTTGC	Up
*acc* (6731)	GCCAAAACACATTATCAGGAG	GATGTTCTTGCCTACAGTAAGTCCG	Up
*gpdh* (6629)	GAATGGGGGACCTCATCGTCACTGC	CAGTCTTTTCACGCATCATCAGGGC	Up
*shmt* (7731/7730)	CTTACCACTGAAGTTGACTGGGCTG	CTGAAGGCTATCCCCACAAACG	Up
*ftsZ* 1(613)^b^	ACGGCAAGCAAGTAGAATCCGAGGC	GGTCTACAAATGGACACACCACGGG	Up
*ftsZ* 2(613)^b^	TCACCACTGGAGACCCCGATGCC	GTCATCCCGAACGACCGTCTCCTGG	
*hp1* (213)	ATCACAGACCGCAAGGATGTAACC	CCACAATCAGTTCACGGACAACAGG	Up
*clp* (5114)	CGTAGCGAACTCCGTCGTCAGCCAG	TTTCTATTTCTGTCGCCTGACCCTG	Up
*gluRS* (6812)	TCGGCACACGGAAACGGATGCTTGG	CGGAACGCCAGCACATCTACAAGCC	Up
*cys*1 (5417)^b^	CGGTCTTGTATCCCTTGCTTGCTGC	GTCATCGGAAACACGCCACTCG	Up
*cys*2 (5417)^b^	GCTGTCGCCACAAAGGCATCG	GAGCGTATCAATCTGCTGAAGGCG	
*pdxk* (5335)	GCACAGATTGCGACAGCACTTTC	GCCTTTGGAATGGATGACTTCTGCG	Up
*nads* (1411)	CTTGTTGTAGCGGTGGTAAGGGAGG	CCCCTGAGCACCTGGCAAATAAG	Up
*hpr* (28)	TCTCATTACAGACGAAACAGG	TCAGTCGGTCAGTCCTTCTTTG	Up
*ndk* (1036)	TCCAAAGGAACTTGGTAGGCACAATC	CACGGATGGTTCCTGGAGCCGCCTG	Up
*sdh* 1(2324)^b^	GATTACTCTGTCCACACCATCTGC	CAGGGTGACGACCATACTCGGGAAC	Up
*sdh* 2(2324)^b^	ACAAGGTCAGTTCACCGCTCAGTCC	CGGCAGCAGAGGACAAGGCGATTTC	
*ult* (8509)	GAGAAAACTCCCCTTTGCGGTCG	TGGGACGGCTGTGCCTGGTCAATG	Up
*fbpa* (2345)	TACAACTTCCTTGATGCCATTTTC	ACACGCATCATCACTTCCCCTTC	Up
*taldo* (7207)	GCCCTCCTCCGTCATTGGAATCTTG	GAGAAATAAATGAGTGGGGTGTGC	Down
*hp2* (8232)	GAAATCCAGAGGGTGGCAATAGGTC	CAGGACACTGACACCCGAACATACG	Down
16s rRNA^c^	TCTCCAGGGTGGTCAGAGGATGTC	TGGAGGAACACCAGTGGCGAAGGC	—

^a^The number in brackets are the same as indicated in the Fig. 3.

^b^The associated protein is encoded by 2 genes, according to the NCBI database.

^c^Internal control gene.
